# *Semecarpus anacardium* (Bhallataka) Alters the Glucose Metabolism and Energy Production in Diabetic Rats

**DOI:** 10.1155/2011/142978

**Published:** 2010-09-08

**Authors:** Jaya Aseervatham, Shanthi Palanivelu, Sachdanandam Panchanadham

**Affiliations:** ^1^Department of Medical Biochemistry, Dr. ALM Post Graduate Institute of Basic Medical Sciences, University of Madras, Taramani Campus, Chennai 600 113, India; ^2^Department of Pathology, Dr. ALM Post Graduate Institute of Basic Medical Sciences, University of Madras, Taramani Campus, Chennai 600 113, India

## Abstract

Glucose produced by gluconeogenesis and glycogenolysis plays an important role in aggravating hyperglycemia in diabetes, and altered mitochondrial function is associated with impaired energy production. The present study focuses on the effect of *Semecarpus anacardium* on carbohydrate metabolism and energy production in diabetic rats. Diabetes was induced by the administration of Streptozotocin at a dose of 50 mg/kg.b.wt. Three days after the induction, *Semecarpus anacardium* at a dose of 300 mg/kg.b.wt was administered for 21 days. After the experimental duration, the activities of the enzymes involved in Glycolysis, TCA cycle, gluconeogenesis, and glycogen were assayed in the liver and kidney of the experimental animals. In addition, to the complexes the protein expression of AKT and PI3K were assayed. The levels of the enzymes involved in Glycolysis and TCA cycle increased, while that of gluconeogensis decreased. The activities of the mitochondrial complexes were also favorably modulated. The expressions of PI3K and AKT also increased in the skeletal muscle. These effects may be attributed to the hypoglycemic and the antioxidative activity of *Semecarpus anacardium*. The results of the study revealed that *Semecarpus anacardium* was able to restore the altered activities of the enzymes involved in carbohydrate metabolism and energy production.

## 1. Introduction

Diabetes mellitus is a metabolic disorder characterized by hyperglycemia due to defect in insulin secretion, action, or both. Liver plays an important role in the glucose homeostasis through glycolysis, glycogenesis, and gluconeogenesis. The net glucose uptake by the liver depends on the activities of glucokinase and glucose 6 phosphatase. The activity of hepatic glucokinase is markedly decreased and activity of glucose-6-phosphatase is almost doubled [1]. Glucose, taken up by secondary active transporter proteins, is degraded to pyruvate, which is then introduced into the citric acid cycle after its decarboxylation to acetyl coenzyme A. The Krebs cycle provides NADH for oxidative phosphorylation to generate the electron gradient for ATP formation. 

Medicinal plants have played a significant role in various ancient traditional systems of medicine. They are rich sources of bioactive compounds and thus serve as important raw materials for drug production and have become a target for the search of new drugs [2]. *Semecarpus anacardium* L. (Anacardiaceae) (SA) commonly known as Bhallataka or marking nut is used in indigenous systems of medicine for the treatment of various diseases [3]. Many compounds mainly biflavonoids, phenolics, bhilawanols, sterols, Anacardic acid, and glycosides have been identified as constituents of *S*. *anacardium* nut extract. On the basis of chemical and spectral data, several biflavonoids, such as Jeediflavanone, Galluflavanone, Nalluflavanone, Semecarpetin, and Anacardiflavanone have been characterized [4]. Several monophenolic compounds known as Semecarpol (C17H28O) and Bhilawanol were also isolated [5]. The drug is used as milk extract to treat many diseases as detailed by many texts specially Caraka Samhita [6]. Earlier studies from our laboratory have proved the presence of poly phenols in the nut milk extract [7]. TLC, HPLC, and HPTLC analysis of the nut and milk extract confirmed the presence of the above compounds [8–11]. The kernel oil contains oleic acid, 60.6; linoleic acid, 17.1; palmitic acid, 16; stearic acid, 3.8; arachidic acid, 1.4%. Studies have also reported that the drug has anti-inflammatory, antiarthritic, anthelmentic, antioxidative and anticancer activity [12, 13]. The nut milk extract has anticancer, hepatoprotective activity, anti-inflammatory, antioxidant property [14], and hypoglycemic activity [15]. Since Insulin suppresses hepatic glucose output by stimulating glycogen synthesis and inhibiting glycogenolysis and gluconeogenesis, a drug that would stimulate the insulin production or sensitize the peripheral tissues to insulin would be of beneficial effect to treat Diabetes Mellitus. The holistic medical approach provided by traditional medicine prompted us to undertake the present study to prove the effect of SA on the altered activities of carbohydrate-metabolizing enzymes and energy production in diabetic rats. The effect of SA on the expression of PI3K and AKT was also investigated.

## 2. Material and Methods

Male Albino rats of Wister strain weighing 260 ± 10 g were used in this study. The animals were housed in polypropylene cages under a control environment with 12 h light/dark cycles and a temperature between 27 and 37°C and were given a commercial diet with water ad libitum. All experiments involving animals were conducted according to NIH guidelines, after obtaining approval from the Madras Institute's Ethical Committee IEAC no 02/075/06. The milk extract of SA was prepared according to the Formulary of Siddha Medicine, by boiling the nuts (200 g) with 500 mL milk. Decanting the decoction, 500 mL of milk was added to the boiling nuts and again boiled for some time. The decoction was recovered and the process was repeated again with the milk (500). All the three portions of milk nut decoction were mixed with ghee (1.5 kg) and boiled till dehydration. Then it was filtered and stored. Olive oil was used as a vehicle for the suspending of the extract and administering to rats [16].

## 3. Experimental Design

Male albino Wister rats weighing 250–270 g were divided into five groups of six animals each (see Table 1).

### 3.1. Biochemical Analysis

After the experimental period, the animals were killed by cervical decapitation. The liver, kidney, and skeletal muscle were excised immediately and immersed in ice-cold physiological saline. Ten per cent homogenate was prepared with fresh tissue in 0.01 M Tris-HCl buffer (pH 7.4) and was used for the following assays. Hexokinase was assayed by the method of Brandstrup et al. [17]. Phosphogluco-isomerase was assayed by the method of Horrocks et al. [18]. Phosphofructokinase was assayed kinetically by the method of Reinhart and Lardy [19]. Aldolase was estimated by the method of King [20]. LDH was assayed by the method of King [21]. Glucose-6-Phosphate Dehydrogenase was assayed by the method of Beutler and West [22]. Glucose-6-phosphatase and Fructose 1, 6-bisphosphatase were assayed according to the method of J. M. Gancedo and C. Gancedo [23]. Glycogen in liver and kidney was estimated by the method of Morales et al. [24].

Mitochondria were isolated by the method of Johnson et al. [25]. Isocitrate Dehydrogenase was assayed according to the method of [20]. The activity of SDH was assayed according to the method of Slater and Borner Jr. [26]. Malate Dehydrogenase was assayed by the method of Mehler et al. [27]. *α*-ketoglutarate dehydrogenase was assayed by the method Reed and Mukherjee [28]. Complex I activity was measured by following the decrease in absorbance due to oxidation of NADH to NAD at 340 with 425 nm as the reference wavelength by the method of Hatefi [29]. Complex II activity was measured by following the decrease in absorbance due to coupled reduction of 2,6-dichlorophenolindophenol (DCPIP) at 60 mM with 750 nm as the reference wavelength [30]. Complex III activity was measured by following the increase in absorbance due to reduction of ferricytochrome c at 550 with 580 nm as the reference wavelength (*ε* = 19 mM^−1^ cm^−1^) [31]. Complex IV activity was measured by following the oxidation of cytochrome c Fe2+ [32]. 

### 3.2. Western Blot Analysis of PI3K and AKT in Skeletal Muscle of Control and Experimental Animals

The protein concentration of the skeletal muscle for PI3K, AKT was estimated and the samples (equal amount of protein; 50 *μ*g) were boiled with sample solubilizing buffer (SSB) for 5 min and separated on 10% sodium dodecyl sulfate-polyacrylamide gel electrophoresis (SDS–PAGE). The gel was transferred onto a nitrocellulose membrane (Hybond C+, Amersham life sciences) at 30 V for 5 h. Membrane was then washed thrice with PBS and blocking was done with TBST buffer (20 mM Tris, 500 mM NaCl, and 0.1% Tween 20, pH 7.5) containing 5% nonfat dry milk. Then, the membrane was incubated with primary antibody (rabbit polyclonal anti-PI3K) and AKT (mouse) in TBST buffer containing 1% nonfat dry milk and agitated gently at room temperature for 3 h. After incubation with the primary antibody, the blots were washed thrice for 5 min with TBST buffer and incubated for 75 min at room temperature with horseradish peroxidase (HRP) conjugated secondary antibody (1 : 500 dilutions) in phosphate-free TBST buffer containing 5% nonfat dried milk. The bands were detected using DAB/hydrogen peroxide chromogen system.

### 3.3. Statistical Analysis

The values are expressed as mean ± SD for six rats in each group. Statistically significant differences between the groups were calculated using one-way analysis of variance (ANOVA), followed by student-Newman-Keuls for multiple comparisons using statistical package for social sciences (SPSS) computer package. Values of *P* < .05 were considered to be significant.

## 4. Results

### 4.1. Activities of Glycolysis and Gluconeogenic Enzymes

**Figure 1:**
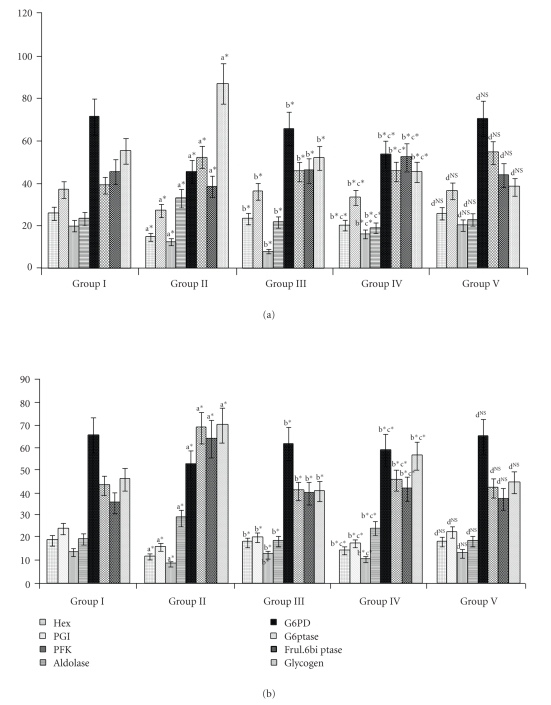
(a) Glycolytic and gluconeogenic enzymes in liver of control and experimental animals. Units: Hexokinase—nmoles of glucose-phosphate liberated/min/mg protein, Phosphoglucoisomerase—nmoles of fructose liberated/min/mg protein, Phosphofructokinase—nmoles of substrate formed/min/mg protein, Aldolase—nmoles of glyceraldehyde liberated/min/mg protein, Glucose 6-phosphatase—nmoles of phosphorous liberated/min/mg protein, Fructose-1,6-diphosphatase—nmoles of phosphorous liberated/min/mg protein. Glycogen—mg/g tissue. Values are expressed as mean ± SD for six animals. Comparisons are made between “a”—Group II versus Group I, “b”—Group III and IV versus Group II, “c”—Group IV versus Group III, and “d”—Group V versus Group I. The symbol ∗ represents the statistical significance at *P* < .05, NS—Nonsignificant. (b) Glycolytic and gluconeogenic enzymes in the kidney of control and experimental animals. Units: Hexokinase—nmoles of glucose-phosphate liberated/min/mg protein, Phosphoglucoisomerase—nmoles of fructose liberated/min/mg protein, Phosphofructokinase—nmoles of substrate formed/min/mg protein, Aldolase—nmoles of glyceraldehyde liberated/min/mg protein, Glucose 6-phosphatase—nmoles of phosphorous liberated/min/mg protein, Fructose-1,6-diphosphatase—nmoles of phosphorous liberated/min/mg protein. Glycogen—mg/g tissue.Values are expressed as mean ± SD for six animals. Comparisons are made between “a”—Group II versus Group I, “b”—Group III and IV versus Group II, “c”—Group IV versus Group III, and “d”—Group V versus Group I. The symbol ∗ represents the statistical significance at *P* < .05, NS—Nonsignificant.

The activities of the carbohydrate-metabolizing enzymes in liver and kidney are given in Figures 1(a) and 1(b). In Group II animals, there was a significant decrease (*P* < .05) in the activities of Hexokinase, phosphoglucoisomerase, and phosphofructokinase in the liver and kidney. The values decreased by 38% for hexokinase, 33.6% for phosphoglucoisomerase, and 39.5% for phosphofructokinase in liver, and in the kidney the activities decreased by 44.4% for hexokinase, 27% for phosphoglucoisomerase, and 39% for phosphofructokinase. These were significantly increased (*P* < .05) upon drug administration (Group III and Group IV).

The activities of aldolase increased (*P* < .05) significantly in untreated diabetic animals (Group II) which were reverted back to near normal levels upon SA (Group III) and Metformin (Group IV) treatment. A marked decrease in the activity of G6PDH was seen in the liver and kidney for Group II animals. In liver, the activity decreased by 57.5%, and in kidney, the activity decreased by 29.1%. These were significantly increased (*P* < .05) in the SA and Metformin-treated groups (Group III and Group IV). Nonsignificant values were obtained when Group I and Group V animals were compared.

The activities of Fructose 1,6 bis phosphatase (FBPase) and glucose-6-phosphatase (G-6pase) increased in liver and kidney of diabetic untreated animals (Group II). There was 1.92-fold increase in FBPase in liver and G-6Pase increased by 1.3-fold. In the kidney, the fold increase was 1.7 and 1.3 for both the enzymes, respectively. These decreased significantly (*P* < .05) upon SA and Metformin treatment. Nonsignificant values were obtained when the the Metformin treated (Group IV) and and SA treated (Group III) were compared. No significant changes were obtained when Group I and Group V animals were compared.

The glycogen content was found to be decreased in the liver (*P* < .05) and increased (*P* < .05) in the kidney of Group II diabetic animals. These were reverted back to near normal levels on administration of SA. When Group III and Group IV animals were compared, SA was found to be more effective than Metformin. No significant changes were observed when Group I and Group V animals were compared.

**Figure 2:**
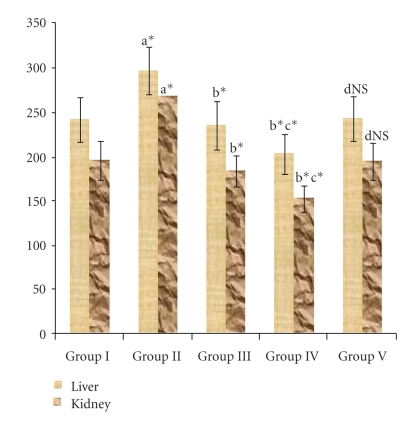
LDH activity in the liver and kidney of control and experimental animals. Lactate dehydrogenase—*μ*moles of pyruvate formed/min/mg protein.

The activities of LDH increased (*P* < .05) significantly in untreated diabetic animals (Group II) which were reverted back to near normal levels upon SA (Group III) and Metformin (Group IV) treatment (Figure 2).

### 4.2. Modification of Energy Production by SA

**Figure 3:**
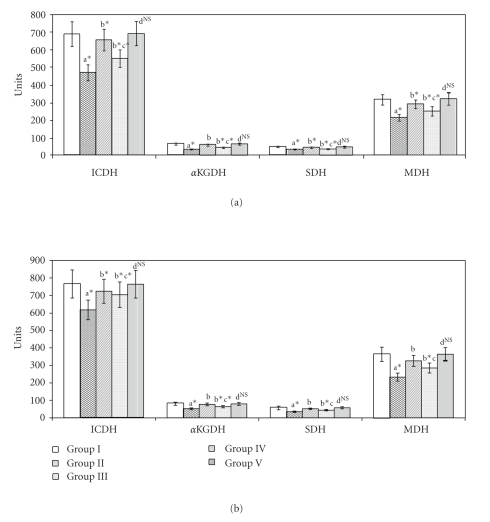
(a) Activities of Citric acid cycle enzymes in liver of control and experimental animals. Units: ICDH—nmol of *α*-ketoglutarate liberated/min/mg protein, *α*-KDH—*μ*mol of ferrocyanide liberated/min/mg protein, SDH—*μ*mol of succinate oxidized/min/mg protein, MDH—nmol of NADH oxidized/min/mg protein. Values are expressed as mean ± SD for six animals. “a”—Group II versus Group I, “b”—Group III and IV versus Group II, “c”—Group IV versus Group III, “d”—Group V versus Group I. The symbol ∗ represents the statistical significance at *P* < .05, NS—Nonsignificant. (b) Activities of Citric acid cycle enzymes in liver of control and experimental animals. Units: ICDH—nmol of *α*-ketoglutarate liberated/min/mg protein, *α*-KDH—*μ*mol of ferrocyanide liberated/min/mg protein, SDH—*μ*mol of succinate oxidized/min/mg protein, MDH—nmol of NADH oxidized/min/mg protein. Values are expressed as mean ± SD for six animals. “a”—Group II versus Group I, “b”—Group III and IV versus Group II, “c”—Group IV versus Group III, “d”—Group V versus Group I. The symbol ∗ represents the statistical significance at *P* < .05, NS—Nonsignificant.

The effects of SA and Metformin on the activities of TCA cycle enzymes in the kidney and liver are given in Figures 3(a) and 3(b). In Group II animals, the activities of ICDH, *α*KGDH, SDH, and MDH were found to be decreased in the liver and kidney. The decrease for ICDH was 19.5% in liver and 16.2% in kidney, *α*KGDH; 35.4% and 34.6% in liver and kidney, SDH; 29.9% and 34.6% for liver and kidney, and for MDH it was 30% and 26% for liver and kidney, respectively. These values were significantly increased (*P* < .05) in Group III and Group IV animals. When Group I and Group V animals were compared, nonsignificant values were obtained.

**Figure 4:**
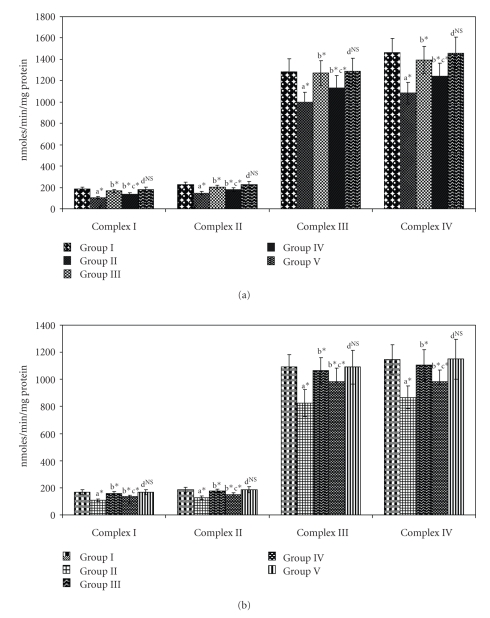
(a) Effect of SA on the activities of mitochondrial complexes in liver of control and experimental animals. Values are expressed as mean ± SD for six animals. “a”—Group II versus Group I, “b”—Group III and IV versus Group II, “c”—Group IV versus Group III, “d”—Group V versus Group I. The symbol ∗ represents the statistical significance at *P* < .05, NS—Nonsignificant. (b) Effect of SA on the activities of mitochondrial complexes in kidney of control and experimental animals. Values are expressed as mean ± SD for six animals. “a”—Group II versus Group I, “b”—Group III and IV versus Group II, “c”—Group IV versus Group III, “d”—Group V versus Group I. The symbol ∗ represents the statistical significance at *P* < .05, NS—Nonsignificant.

In untreated diabetic animals (Group II), marked inhibition (*P* < .05) of the activities of mitochondrial complexes was found as shown in Figures 4(a) and 4(b) when compared to normal animals. These were restored to near normal levels (*P* < .05) upon drug administration (Group III and Group IV). No significance was found when control (Group I) and drug control group (Group V) animals were compared.

### 4.3. SA Increases the Protein Expression on PI3K and AKT in the Skeletal Muscle of Control and Experimental Animals

**Figure 5:**
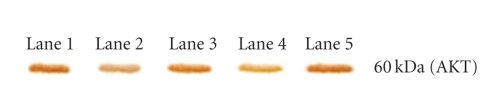
Western blot analysis of AKT in skletal muscle of control and experimental animals. Lane 1—control, Lane 2—induced, Lane 3—SA treated, Lane 4—Metformin treated, Lane 5—drug control.

**Figure 6:**
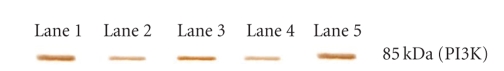
Western blot analysis of PI3K in skeletal muscle of control and experimental animals. Lane 1—control, Lane 2—induced, Lane 3—SA treated, Lane 4—Metformin treated, Lane 5—drug control.

The protein expressions of AKT and PI3K are shown in Figures 5 and 6. In Group II animals, there was decrease in protein expression of PI3K and AKT when compared to Group III, indicating the insufficiency of insulin to maintain the normal signaling and the uptake of glucose through GLUT4 in the muscles of the animals which results in hyperglycemia. After treatment of the animals with SA, increase in protein expression was seen. 

## 5. Discussion

Liver plays a key role in the maintenance of glucose homeostasis. Following a carbohydrate rich meal, it removes a major part of the excess glucose that is absorbed and releases it in between meals during starvation and exercise [33]. The glucostat function of the liver is based on the reversible shift between glycogen synthesis and degradation as well as between glycolysis and gluconeogenesis. 

The reaction catalyzed by hexokinase serves as the entry point for glucose into glycolysis, glycogen synthesis, and the hexose monophosphate. Inhibition of hexokinase leads to impaired oxidation of glucose via glycolysis, resulting in hyperglycemia and decreased ATP production [34]. High glucose concentration nonenzymatically glycates phosphoglucoisomerase and inhibits the proportion of glucose 6-phosphate metabolized via the glycolytic pathway. Insulin influences the intracellular utilization of glucose by increasing the activity and amount of several key enzymes including glucokinase and phosphofructokinase. Increased Aldolase seen in untreated diabetic rats may be due to cell impairment and necrosis. Administration of SA enhanced the uptake of glucose and increased glycolysis [35]. Oxidative stress seen in diabetes may be responsible for the increase in LDH. The release of LDH reflects a nonspecific alteration in the plasma membrane integrity and/or permeability as a response to STZ. The decreased level of LDH in SA-treated group is due to the presence of poly phenols which have strong antioxidative property [14]. Insulin increases Glucose-6-phosphate dehydrogenase (G6PDH) activity of rat liver cells, while high glucose inhibits it. The observed decrease in G6PDH activities may be due to the reduced insulin secretion and action, or inhibition of this enzyme due to phosphorylation or oxidative modification. G6PDH protects the cell from death, which is inactivated by lipoperoxidation products such as 4-hydroxy-2-nonenal [36]. SA increases the activity of G6PDH in the Group III animals by decreasing the lipid peroxidation [35]. The elevated hepatic glucose production (HGP) seen in Group II animals is due to increased rate of gluconeogenesis rather than glycogenolysis [37]. In Type I Diabetes, the mRNA levels of Glucose 6 phosphatase catalytic subunit and activity are increased which lead to increased HGP [38]. Increased hepatic glucose production in Diabetes Mellitus is also associated with impaired suppression of fructose-1,6-bisphosphatase [39]. In the diabetic state, the activities of the glycolytic enzymes are decreased with increase in gluconeogenic enzymes as seen in Group II animals. These were effectively reverted back to near normal levels upon administration of SA [40]. The low glycogen content in diabetic rat liver could increase glucose uptake and hexokinase activity, resulting in an increase in intracellular Glucose 6 phosphate concentration and consequently in an increase in Glycogen synthase activity. The renal hypertrophy observed is closely correlated with the degree of hyperglycemia. It is possible, therefore, that there might have been the breakdown of this glycogen that could have contributed to the increase in renal glucose release [41].

Glucose metabolism, the citric acid cycle, and oxidative phosphorylation are central biochemical pathways in cellular energy metabolism. Hyperglycemia glycates ICDH which results in reduced activity [42]. *α*-KGDH could be a crucial target of reactive oxygen species (ROS) and being an important regulatory site in the mitochondrial metabolism could play a key role in the bioenergetic deficit evolving oxidative stress. One of the mitochondrial proteins selectively targeted by 4-Hydroxynonenal (HNE) is the FAD-containing subunit of SDH. This result is in good agreement with the decrease in oxygen consumption in the presence of succinate and in complex II activity as reported by Lashin et al. [43]. The decreased activities of the citric acid cycle enzymes in diabetic rats were brought back to near normal levels upon administration of the drug, which may be due to the positive modification of the SDH subunit [44] and its hypoglycemic effect [45]. Many of the ETC complex subunits appear to be specific targets for ROS-mediated oxidative modifications. Mitochondria isolated from Type I Diabetic rats showed decreased respiratory chain activity characterized by decreased membrane potential, decreased expression of oxidative phosphorylation genes, and respiratory ratios. The mRNAs for Complex I, II, III, and IV were also found to be decreased [46]. Munusamy et al. reported kidney mitochondrial impairment in diabetes. The decline in renal mitochondrial respiration is due to increased ROS production at complexes I, III, and IV [47]. SA normalized the activities of the complexes and decreased the production of superoxide due to its radical scavenging activity, which may be attributed to the flavonoids and other constituents present in the drug.

Membrane-associated signaling processes are a critical part of the complex pathways that transduce insulin-mediated change in cellular metabolism. After insulin binds to its receptor, activation of at least two major pathways, one involving a ras/mitogen-activated protein kinase (MAPK) cascade and the other involving phosphatidylinositol-3-kinase (PI3-kinase) takes place. PI 3-kinase is well recognized as an important step in the insulin signaling pathway to glucose transport through GLUT4 translocation [48]. 

Activated PI3K activates downstream component Akt (PKB). Overexpression of constitutively active AKT directly promotes glucose transport and translocation of GLUT1 and GLUT4 to the plasma membrane. Insulin increases the association of AKT with GLUT4-containing vesicles. Increased expression of PI3K and AKT in the SA-treated group may be due to the presence of polyphenols since they have been implicated in mediating glucose transport *in vitro *and *in vivo *studies [49].

The protective effect of SA may be due to its antioxidative [14] and hypoglycemic activity [45]. These could be attributed to the presence of flavonoids. Flavonoids are a group of ubiquitously distributed plant polyphenols which exhibit a wide range of pharmacological effects. The inhibition of lipid peroxidation is due to the free radical scavenging property of flavonoids. They scavenge free radical by donating their hydrogen groups and prevent the initiation of chain reaction. They also scavenge singlet O_2_, terminating peroxides by their low redox potential [50]. They also reduce lipid peroxidation by reducing the levels of malondialdehyde and conjugated dienes [51]. The antioxidative activity of the SA is due to the presence of flavonones, present in the drug which contains a catechol moiety in the B ring. In the Galluflavanone, the catechol moiety in the secondary flavanone ring also confers the antioxidant property. The 4-oxo group present in all the biflavanones in SA contributes to the free radical quenching activity. The 5-OH and 7-OH groups present in Jeediflavanone also confer scavenging potential. Semecarpuflavanone, Semecarpetin, and Galluflavanone have a 7-OH group that might contribute to the free radical-chelating activity of SA [52].

## 6. Conclusions

From the above observations it can be concluded that SA is able to favorably modulate the activities of the enzymes involved carbohydrate metabolism. It was also able to restore the altered activities of the TCA cycle enzymes and normalize the alteration in energy production and increase the expression of PI3K and AKT in the skeletal muscles leading to increase in the uptake of glucose by the cells. 

## Figures and Tables

**Table 1:** 

Group I	Control animals—Normal healthy controls received olive oil (0.5 mL) orally by gastric intubation for 21 days daily
Group II	Diabetes induced—(50 mg/kg b.wt.) Streptozotocin dissolved in 0.5 mL of 0.1 M citrate buffer PH 4.5.
Group III	SA treated—Three days after the induction of diabetes, SA (300 mg/kg body weight dissolved in 0.5 mL olive oil) was administered by gastric intubation for 21 days daily.
Group IV	Metformin treated—Three days after the induction of diabetes Metformin (500 mg/kg body weight dissolved in 0.5 mL physiological saline) was administered by gastric intubation for 21 days daily.
Group V	Drug control—Animals received SA at a dose of 300 mg/kg b. wt. in olive oil (0.5 mL) orally by gastric intubation for 21 days daily.
